# Irradiation of rat brain reduces P-glycoprotein expression and function

**DOI:** 10.1038/sj.bjc.6603864

**Published:** 2007-07-03

**Authors:** J Bart, W B Nagengast, R P Coppes, T D Wegman, W T A van der Graaf, H J M Groen, W Vaalburg, E G E de Vries, N H Hendrikse

**Affiliations:** 1 Department of Pathology, University of Groningen and University Medical Center Groningen, P.O. Box 30.001, 9700 RB, Groningen, The Netherlands; 2 Department of Medical Oncology, University of Groningen and University Medical Center Groningen, P.O. Box 30.001, 9700 RB, Groningen, The Netherlands; 3 Department of Radiation and Stress Cell Biology, University of Groningen and University Medical Center Groningen, P.O. Box 30.001, 9700 RB, Groningen, The Netherlands; 4 Department of Nuclear Medicine and Molecular Imaging, University of Groningen and University Medical Center Groningen, P.O. Box 30.001, 9700 RB, Groningen, The Netherlands; 5 Department of Pulmonology, University of Groningen and University Medical Center Groningen, P.O. Box 30.001, 9700 RB, Groningen, The Netherlands

**Keywords:** blood–brain barrier, P-glycoprotein, [^11^C]carvedilol, irradiaton

## Abstract

The blood–brain barrier (BBB) hampers delivery of several drugs including chemotherapeutics to the brain. The drug efflux pump P-glycoprotein (P-gp), expressed on brain capillary endothelial cells, is part of the BBB. P-gp expression on capillary endothelium decreases 5 days after brain irradiation, which may reduce P-gp function and increase brain levels of P-gp substrates. To elucidate whether radiation therapy reduces P-gp expression and function in the brain, right hemispheres of rats were irradiated with single doses of 2–25 Gy followed by 10 mg kg^−1^ of the P-gp substrate cyclosporine A (CsA) intravenously (i.v.), with once 15 Gy followed by CsA (10, 15 or 20 mg kg^−1^), or with fractionated irradiation (4 × 5 Gy) followed by CsA (10 mg kg^−1^) 5 days later. Additionally, four groups of three rats received 25 Gy once and were killed 10, 15, 20 or 25 days later. The brains were removed and P-gp detected immunohistochemically. P-gp function was assessed by [^11^C]carvedilol uptake using quantitative autoradiography. Irradiation increased [^11^C]carvedilol uptake dose-dependently, to a maximum of 20% above non irradiated hemisphere. CsA increased [^11^C]carvedilol uptake dose-dependently in both hemispheres, but more (*P*<0.001) in the irradiated hemisphere. Fractionated irradiation resulted in a lost P-gp expression 10 days after start irradiation, which coincided with increased [^11^C]carvedilol uptake. P-gp expression decreased between day 15 and 20 after single dose irradiation, and increased again thereafter. Rat brain irradiation results in a temporary decreased P-gp function.

The blood–brain barrier (BBB) is a major impediment for the delivery of several drugs to the brain, including cytotoxic, anti-epileptic, and anti-HIV drugs. The BBB is formed by specialised capillary endothelial cells, which have several properties, such as strong tight junctions with a high electrical resistance, absence of fenestrations and presence of efflux-pumps such P-glycoprotein (P-gp) and multidrug resistance associated protein 1 (MRP1). The efflux pumps are transmembrane proteins, able to extrude actively toxic compounds against a concentration gradient. P-gp effluxes a broad spectrum of natural compounds, among which chemotherapeutic drugs, such as anthracyclines, taxanes, and epipodophyllotoxins (Bart *et al*, 2000). MRP1 transports also a broad spectrum of substrates, but these substrates are transported in conjugation with or co-transported with glucuronide or glutathione. Strategies to pass the BBB include therapies to change the physical properties of the capillary endothelium by hypertonic solutions or by bradykinin analogues (Bartus *et al*, 1996; Elliott *et al*, 1996). Other strategies are directed to inhibition of efflux pump function. At present the concept of efflux pump inhibition is most frequently studied for P-gp. Studies in rodents have shown that P-gp function can be inhibited with cyclosporin A (CsA) (Hendrikse *et al*, 1998, 1999; Syvanen *et al*, 2006). To circumvent toxic side effects of CsA in patients, therapies that influence P-gp function could be utilised, such as radiotherapy. Early effects on brain capillary endothelium were studied by Mima *et al* (1999). They exposed one brain hemisphere of rats to a single dose of 25 Gy and demonstrated that P-gp expression was decreased in the irradiated hemisphere compared to the non-irradiated hemisphere 5 days after irradiation. It is therefore hypothesised that brain irradiation could also be used to enhance the delivery of P-gp substrates to the brain. P-gp function can be measured with the aid of radiolabelled P-gp substrates, such as [^11^C]verapamil and [^11^C]carvedilol (Hendrikse *et al*, 1999; Bart *et al*, 2003, 2005; Syvanen *et al*, 2006; Takano *et al*, 2006). Other approaches like the ^111^In-labelled 15D3 monoclonal anti-P-gp has been used to visualise P-gp expression (van Eerd *et al*, 2006). To elucidate whether the decrease in P-gp expression, which was determined with immunohistochemistry using C219, also resulted in a decrease of P-gp function, the latter was assessed by autoradiography using [^11^C]carvedilol (Bart *et al*, 2005). The right brain hemisphere of rats was irradiated with single fractions of radiotherapy. Furthermore, to mimic clinically relevant palliative whole brain radiotherapy, radiation was also administered in five fractions of 4 Gy.

## MATERIALS AND METHODS

### Chemicals

Ketamine (Ketanest® 25 mg ml^−1^) was obtained from Parke-Davis (Munich, Germany), medetomidine (Domitor® 10 mg ml^−1^) from Pfizer (New York, NY, USA), CsA (50 mg ml^−1^) in cremophore EL (650 mg ml^−1^) (Sandimmune®/Neoral®) from Novartis (Basel, Switzerland) and C219, a monoclonal antibody against rat and human P-gp, from Thamer Diagnostica (Uithoorn, The Netherlands). Peroxidase-conjugated rabbit-anti-mouse secondary antibody (RAMPO) and peroxidase-conjugated goat-anti-rabbit antibody (GARPO) were purchased from DAKO (Glostrup, Denmark), 3.3-diaminobenzidine tetrahydrochloride and isopentane from Sigma (St. Louis, MO, USA), phosphate buffered saline (PBS, 140 mmol l^−1^ NaCl, 9 mmol l^−1^ Na_2_HPO_4_, 9 mmol l^−1^ NaH_2_PO_4_; pH 7.4)) and imidazole from Merck (Darmstadt, Germany), and bovine serum albumin (BSA) from Serva Electrophoresis GmbH (Hamburg, Germany). Desmethyl carvedilol was kindly provided by Roche (Mannheim, Germany).

### Preparation of [^11^C]carvedilol

[^11^C]carvedilol was synthesised as described earlier (Doze *et al*, 2002), with some modifications. Desmethyl carvedilol reacts with [^11^C]methyltriflate, in the presence of K_2_CO_3_ (4 mg) with kryptofix (4 mg) in 400 *μ*l dry acetone for 5 min at 85°C. The product was purified by HPLC Platinum C_18_ column (300 × 7.8 mm, Alltech, Deerfield, IL, USA) with a solvent system of 25 mM NaH_2_PO_4_ (pH 7.6) : MeOH (43 : 57), flow rate 3 ml min^−1^ and UV detection at 254 nm. After evaporation of the solvent under reduced pressure, [^11^C]carvedilol was dissolved in 0.3 ml saline.

### Animal experiments

Male Wistar rats (HSD CPB wu, Harlan, The Netherlands) weighing 225–275 g, were anesthetised with an intra-peritoneal injection of a mixture of S-ketamine 25 mg ml^−1^ and medetomidine 1 mg ml^−1^ (5 : 1, 1 ml kg^−1^). However, the rats who underwent fractionated irradiation were anesthetised with inhaled isoflurane/oxygen. The irradiations were performed with a Philips/Muller MG 300 X-ray machine operated at 200 kV (HVL=1.05 mm Cu) and a beam of 15 mA. The rat was protected against X-rays with a 3 mm lead shield, except the right half of the skull. The right hemisphere was irradiated with a dose rate of 1 Gy min^−1^ as measured in air with a calibrated electrometer and ionisation chamber combination (Keithley 35040+NE 2571) at source to skin distance of 32 cm.

Four groups of rats were formed: Group 1 received 2 (*n*=4), 5 (*n*=4), 10 (*n*=4), 15 (*n*=4), and 25 Gy (*n*=5) once, group 2 received 15 Gy once, group 3 received fractionated irradiations (5 days 4 Gy, biologically effective dose equal to ≈10 Gy when an *α*/*β* of 2 for brain is used) (van der Kogel, 2002) and group 4 received 25 Gy (*n*=12) once. Five days post-irradiation, all rats were anesthetised again with the medetomidine/S-ketamine mixture, and the tail was cannulated. Rats in groups 1 and 3 received 10 mg kg^−1^ CsA, to obtain a better signal/noise ratio, and in group 2 10 mg kg^−1^ (*n*=4), 15 mg kg^−1^ (*n*=4), 20 mg kg^−1^ (*n*=4) CsA. CsA was administered intravenously (i.v.) after 30 min followed by [^11^C]carvedilol i.v. After 15 min the rats were killed by extirpation of the heart and the brain was removed. One part of the brain was fixed in formalin and embedded in paraffin for immunohistochemistry and the second part was frozen in isopentane at −70°C for autoradiographic assessment. The rats in group 4 were killed 10, 15, 20, and 25 days after irradiation. The removed brains were fixed in formalin, embedded in paraffin, and stained for P-gp.

Animal studies were conducted according to the UKCCCR Guidelines for the care and use of laboratory animals.

### Histology/Immunohistochemistry

The embedded brains were cut into 4 *μ*m slices, placed on positively charged glass slides, and dried. The slices were dewaxed in xylene and re-hydrated in serial ethanol washes (100, 96, and 70%). Subsequently, they were washed three times in 1% BSA/PBS (pH 7.4). For histology, the slices were stained with hematoxylin/eosin. For immunohistochemistry, the slices were heated three times for 10 min in an autoclave at 115°C in 20 mM blocking reagens (Boehringer Mannheim, Mannheim, Germany) at pH 6.0. Endogenous peroxidase was blocked by incubation with 1% H_2_O_2_ in PBS during 30 min and a-specific antigens were blocked with 1% rat serum in PBS. The slices were incubated with C219 antibody in 1% BSA/PBS (pH 7.4) for 1 h at room temperature in a humidified chamber. The primary antibody was detected with a peroxidase conjugated rabbit anti-mouse secondary antibody (1 : 50 diluted in 1% BSA/PBS) followed by incubation with peroxidase labelled goat-anti-rabbit antibody (1 : 50 diluted in 1% BSA/PBS). As chromagen served 3,3-diaminobenzidine tetrahydrochloride (25 mg) and imidazole (50 mg) in PBS (50 ml) with 50 *μ*l 30% H_2_O_2_. After counterstaining with Mayer's hematoxylin for 2 min, slices were dehydrated through graded ethanols (70, 96, and 100%) and mounted with coverslips. Rat liver served as positive controls for P-gp staining. A negative control was made by processing a rat liver slice identical to other slices, but without C219 antibody. Slides were scored by two independent investigators, P-gp expression was assessed semi-quantitatively by using a scale of 0–3 (0: no staining, 1: very weak staining, 2: intermediate staining, 3: strong staining). Strong staining was defined as comparable to the strongest stained slide of all tissue slides. Samples were considered negative if less than 10% of a specific subtype of cells was stained.

### Uptake of [^11^C]carvedilol in the brain

Brain slices of 80 *μ*m thickness were prepared with a Cryo-cut microtome, model 840 (American Optical Corp., Buffalo, NY, USA) at −7°C, placed on object glasses and dried at room temperature. Cyclone™ (Super Sensitive, Packard Instrument Company Inc., Meriden, CT, USA) storage phosphor screens were exposed to the slices for at least 400 min (20 half-lifes of [^11^C]) and subsequently analysed with Optiquant analysis software (version 03.00, Packard Instrument Company Inc., Meriden, CT, USA). To convert from the photostimulated luminescence (PSL) of the exposed screen into Bequerel (Bq), a calibration line was made during each experiment. Regions of interest were drawn over the right (irradiated) and the left (non-irradiated) hemisphere in all slices. The ratio of [^11^C]carvedilol uptake in the right over the left hemisphere was calculated. Exposition in each area was quantified with the aid of a calibration line, converted into Bq, and corrected for injected dose of [^11^C]carvedilol and body mass (Murata *et al*, 1996; Sihver *et al*, 1997; Ishiwata *et al*, 1999).

### Calibration

In each experiment a 1 MBq ml^−1^ [^11^C]carvedilol solution in saline was prepared. The solution was diluted to 714, 625, 500, 250, 125, 55.6, 26.3, and 17.2 kBq ml^−1^ and 10 *μ*l of each dilution was loaded on filter paper. The radioactivity (counts/min) of the pieces of filter paper was measured in the *γ* counter (Compu Gamma, LKB Wallace, Turku, Finland) for 15 s, corrected for decay, and converted to Bq. Thereafter, the pieces were placed on the phosphor storage screen at the same time as the brain slices and exposed for the same time. The PSL of the spots from the calibration pieces was measured and corrected for background PSL. The calibration lines were highly reproducible. The mean direction-coefficients of the individual calibration lines was 63 × 10^3^ (s.d.=4.8 × 10^3^, *n*=10). The equation of the linear relationship between PSL and Bq from the combined calibration line was: PSL=65.3 kBq (*r*^2^=0.99). The calibration lines showed a good linearity between the radiation intensity (Bq) and the PSL of the calibration samples of an order of 10 magnitudes.

### Statistics

Wilcoxon rank tests were used to examine paired data from right (irradiated) and left (non-irradiated) hemisphere. Univariate analysis of variance was used to examine differences in uptake ratio of [^11^C]carvedilol between groups of rats, which were exposed to different single doses of X-rays (SPSS, version 12). Significance was defined as *P*<0.05.

## RESULTS

### P-gp function measured by autoradiographic uptake of [^11^C]carvedilol in rat brain

**Figure 1:**
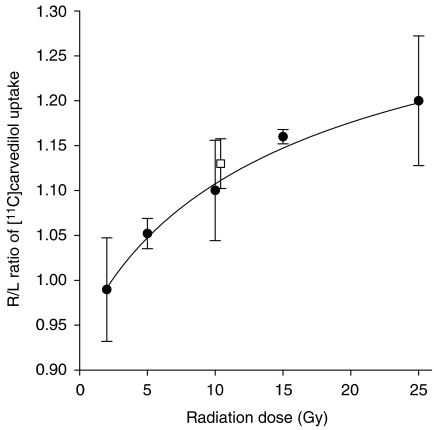
Ratio (±s.d.) of [^11^C]carvedilol uptake in right (irradiated) hemisphere/left (non-irradiated) hemisphere (•). Five days after single-dose irradiation the R/L ratio increases dose-dependently. The fitted curve is a four parameter Hill plot (*r*^2^=0.72). Open square (□) depicts [^11^C]carvedilol accumulation after 5 × 4 Gy irradiation.

The [^11^C]carvedilol uptake in rat brains, expressed as the ratio between the irradiated hemisphere *vs* the non-irradiated hemisphere in group 1, 5 days after different doses of irradiation is shown in Figure 1. Increasing the radiation dose resulted in a higher [^11^C]carvedilol uptake ratio (*P*=0.001). The fitted curve shows a dose-dependent increase of the uptake ratio, to 1.20, which means that the increase in [^11^C]carvedilol uptake by 25 Gy irradiation is 20% compared to the non-irradiated hemisphere. Furthermore, the fitted curve suggests that a plateau of [^11^C]carvedilol uptake will be reached above 25 Gy. In Figure 1 is also depicted that fractionated irradiation for 5 days with 4 Gy (group 3) results in a 13% increased uptake in the irradiated hemisphere, confirming the biologically equal effectiveness to ≈10 Gy single dose for this end point.

**Figure 2:**
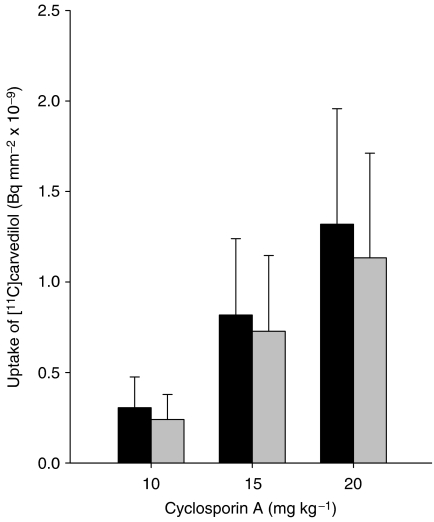
[^11^C]carvedilol uptake (mean±s.d.) in right *vs* left hemisphere after treatment with different dosages of CsA. Note that in individual rats [^11^C]carvedilol uptake is 20–30% higher in the irradiated *vs* non-irradiated hemisphere after 15 Gy (*P*=0.02, Wilcoxon test for paired measures). Interindividual variation masks this effect partly. ▪, irradiated hemisphere (R), ░, irradiated hemisphere (L).

The effect of different doses CsA combined with irradiation was investigated in rats of group 2. Figure 2 shows that increasing dosages of CsA pretreatment resulted in a dose-dependently increased uptake of [^11^C]carvedilol, in both the irradiated and the non-irradiated hemisphere, suggesting that maximal P-gp blockade was not reached. In individual rats, irradiation with 15 Gy single dose resulted in a 20–30% higher uptake in the irradiated hemisphere compared with the non-irradiated hemisphere (*P*=0.02).

### P-gp expression measured by immunohistochemistry

**Figure 3:**
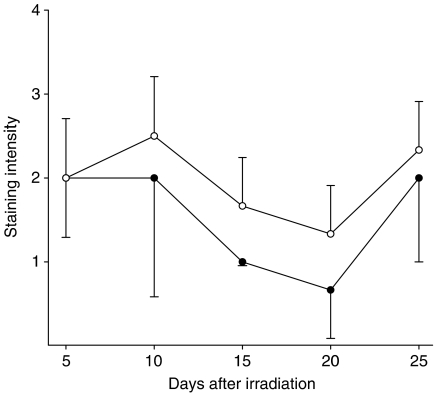
Semiquantitative representation of P-gp expression (mean±s.d.) in non-irradiated hemisphere (○) and irradiated hemisphere (•) at different days after irradiation with 25 Gy, single dose.

**Figure 4:**
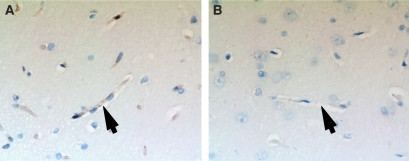
C219 staining (magnification × 400) of a rat brain. Capillary endothelium stains brown in the non-irradiated hemisphere (**A**), revealing the presence of P-gp. In the irradiated hemisphere (**B**), after five fractions of 4 Gy, no P-gp is present in the capillary endothelium. Arrows mark capillaries.

In rats that underwent single-dose irradiation (groups 1 and 2), P-gp was equally expressed at the endothelial wall of brain capillaries in the irradiated and non-irradiated hemisphere on day 5. P-gp expression in the rats that underwent single-dose irradiation (group 4) is shown in Figure 3. P-gp expression is reduced 15 days after irradiation, and this reduction is maximal after 20 days. Interestingly, P-gp expression restores to the same level as before irradiation between days 20 and 25. Furthermore, in rats who underwent fractionated dose irradiation (5 × 4 Gy), P-gp is not detectable immunohistochemically on capillary endothelium 10 days after start of the irradiation (Figure 4A *vs* 4B). As expected other morphological changes of brain tissue were not found at these time points after irradiation.

## DISCUSSION

The present study shows that single and fractionated doses of irradiation of rat brains, increases brain uptake of the P-gp substrate [^11^C]carvedilol after 5 days. This coincided with a maximal reduction in P-gp expression 20 days after single-dose irradiation and a complete loss of P-gp expression 10 days after the start of fractionated irradiation (5 × 4 Gy). After 25 days P-gp expression returned to the same values, as before radiotherapy.

Five days after irradiation there is a discrepancy between P-gp expression and [^11^C]carvedilol uptake. [^11^C]Carvedilol uptake already is higher in the irradiated hemisphere, whereas P-gp expression remains immunohistochemically unaffected. In contrast to Mima *et al* (1999), who showed a decrease in P-gp expression by immunohistochemistry 5 days after irradiation which was quantified by Western blot (−40%), we observed a decrease in P-gp expression between day 15 and 20 after single-dose irradiation. This discrepancy might be explained by the use of different P-gp antibodies. Second, evaluation of P-gp function by assessment of [^11^C]carvedilol uptake is most likely more sensitive compared to semi-quantitative measurement of P-gp expression by immunohistochemistry. Therefore, minor changes in P-gp expression levels might be missed by immunohistochemistry shortly after irradiation, which can already be detected with quantitative PET autoradiography.

We showed that uptake of the P-gp substrate [^11^C]carvedilol already increased 5 days after irradiation, which could indicate that P-gp function decreases earlier than observed with P-gp expression. Interestingly, P-gp expression disappeared completely 5 days after fractions of 4 Gy. Uptake of [^11^C]carvedilol after fractionated irradiation was the same as for the calculated equal biological effective single dose of 10 Gy (van der Kogel, 2002), indicating that a dose-dependent relationship as shown with single-dose irradiation could have clinical implications. The impairment of P-gp functionality after single-dose irradiation is most likely not solely due to a loss of P-gp expression. It is known that irradiation induces membrane damage, which may result in changes in membrane fluidity and in increased apoptosis of irradiated cells (Berroud *et al*, 1996; Giusti *et al*, 1998; Vincent-Genod *et al*, 2001). P-gp function is closely related to membrane fluidity status (Sinicrope *et al*, 1992; Regev *et al*, 1999). Irradiation-induced cell-membrane damage may therefore indirectly cause a loss of function of transmembrane proteins such as P-gp, but could also apply to other efflux pumps in the BBB such as multidrug-resistance-associated proteins, organic anion/cation transporters and glucose transporter 1. Apart from a reduction in P-gp expression, reduced P-gp function may also be due to cell membrane damage, which could be another potential explanation for the discrepancy seen in P-gp function and expression of P-gp 5 days post-irradiation.

Little is known about early effects of irradiation on the BBB. If an early decrease in P-gp expression and P-gp function in the BBB also occurs in patients after brain irradiation, the results of this study may have clinical implications. Since brain irradiation is frequently applied to patients with brain metastases, it can be speculated that irradiation can lead to a temporary window leading to increased delivery of chemotherapeutic drug to the brain. This could be considered as a tool in the treatment and prevention of brain metastases. P-gp expression in the rat brain is maximally reduced 20 days after radiotherapy and decrease of P-gp function can already be measured at day 5 after irradiation. To elucidate whether P-gp function decreases after irradiation in patients, P-gp mediated kinetics can be measured *in vivo* with PET with, for example [^11^C]verapamil as P-gp substrate in patients (Hendrikse *et al*, 1998, 1999; Bart *et al*, 2003, 2005; Syvanen *et al*, 2006; Takano *et al*, 2006). Future studies should elucidate if these results can be extrapolated to the human setting. A study in which patients with brain metastases who receive radiotherapy undergo a [^11^C]carvedilol or [^11^C]verapamil PET scan before and shortly after radiotherapy would give helpful information. In this way an optimal time schedule of chemotherapy following radiotherapy can potentially be determined.
